# Pilot Multi-Matrix Biomonitoring of Mixed Mercury Exposure Pathways Among E-Waste Dismantling Workers in South China

**DOI:** 10.3390/toxics14070584

**Published:** 2026-07-02

**Authors:** Qiyuan Lu

**Affiliations:** 1 Research Center of Emerging Contaminants, South China Institute of Environmental Sciences, Ministry of Ecology and Environment, Guangzhou 510530, China; luqy7@mail3.sysu.edu.cn; 2 The Key Laboratory of Environmental Pollution Health Risk Assessment, South China Institute of Environmental Sciences, Ministry of Ecology and Environment, Guangzhou 510530, China

**Keywords:** electronic waste, mercury, methylmercury, human biomonitoring, exposure assessment, stable isotopes

## Abstract

Informal electronic-waste (e-waste) dismantling can mobilize mercury (Hg) from Hg-containing components and contaminated dust, while local diet can contribute methylmercury (MeHg), creating a mixed environmental exposure setting for human biomonitoring. This pilot study integrated paired biomarkers, local foods, work and indoor dust, Hg speciation and hair Hg isotope signatures among 18 e-waste dismantling workers in Qingyuan, South China. Total Hg (THg), MeHg and inorganic Hg (IHg) were measured in hair, blood and foods; urine and dust were analyzed for THg; and hair δ^202^Hg, Δ^199^Hg and Δ^201^Hg were determined. Median THg was 403 μg/kg in hair, 1.60 μg/L in blood and 0.438 μg/L in urine. Fish showed the highest food THg, whereas work and indoor dust had the highest matrix concentrations. Hair was MeHg-dominant but contained substantial IHg, and Δ^199^Hg values were positive but modest. The integrated patterns support mixed dietary MeHg and non-dietary IHg contributions and identify exposure-pathway priorities for future biomonitoring and source-focused studies.

## 1. Introduction

Electronic waste (e-waste) recycling and dismantling are recognized as potential sources of human exposure to hazardous metals and persistent organic contaminants. In informal or poorly controlled recycling settings, manual disassembly, crushing, heating, open burning and dust resuspension can mobilize contaminants from electronic components and contaminated surfaces. Mercury is of particular concern because it occurs in several electrical and electronic products, including switches, relays, fluorescent lamps, backlights and other Hg-containing components, and because its environmental mobility, transformation, toxicity and exposure pathways depend strongly on chemical form and environmental matrix [1,2].

Human Hg exposure is commonly assessed using biomarkers such as hair, blood and urine, which reflect different exposure windows and Hg species depending on toxicokinetics and exposure route [1,3]. Hair Hg is widely used as an indicator of MeHg exposure, particularly from fish and other aquatic foods, but its interpretation depends on Hg species, external contamination and exposure context [4]. Blood Hg reflects recent exposure to both organic and inorganic Hg, whereas urinary Hg is more closely related to elemental Hg vapor and inorganic Hg exposure, although demethylation of MeHg can also contribute to urinary Hg [5,6]. Therefore, interpretation of Hg biomarkers in occupational or contaminated settings benefits from paired biological matrices, Hg speciation and, where available, isotope information [7].

Several studies have investigated Hg and other metal exposures in e-waste recycling areas. Studies from Guiyu, Taizhou and other South China e-waste regions reported Hg or heavy metals in human hair, food, house dust, indoor dust and other environmental matrices, supporting the importance of multi-matrix exposure assessment in these settings [8,9,10,11,12]. Recent European biomonitoring studies have also examined Hg and other metal exposures among e-waste recycling workers, providing a structured occupational biomonitoring context for this type of exposure assessment [13,14,15]. However, although previous studies separately report biomarker, food/dust, speciation or isotope evidence, limited data are available that combine paired hair, blood and urine biomarkers, local foods, work and indoor dust, Hg speciation and Hg isotope signatures in small dismantling-worker cohorts [10,13,14,16,17].

Hg stable isotopes are increasingly used to distinguish MeHg exposure sources, because positive odd-mass mass-independent fractionation (Δ^199^Hg and Δ^201^Hg) is often associated with aquatic food-web MeHg, whereas rice and terrestrial sources generally show smaller or near-zero MIF signatures [7,18,19,20,21,22]. In China, hair Hg isotope studies have shown that fish- and rice-derived MeHg can produce distinguishable Δ^199^Hg signatures, supporting qualitative or quantitative assessment of dietary MeHg sources in appropriate study designs [18,22].

**Figure 1:**
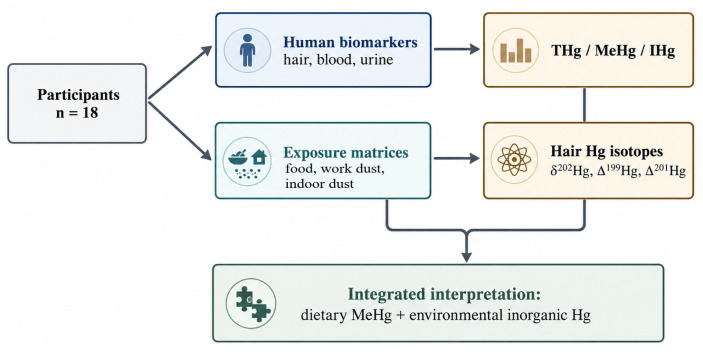
Conceptual design of the pilot multi-matrix biomonitoring study. Human biomarkers, exposure matrices, Hg speciation and hair Hg isotopes were combined to evaluate whether the observed patterns were more consistent with dietary MeHg, environmental IHg, or mixed exposure.

The objective of this pilot study was to characterize Hg exposure pathways among e-waste dismantling workers in South China using paired biomarkers, local foods, work and indoor dust and hair Hg isotopes. The emphasis was placed on integrating environmental sampling, human biomonitoring, Hg speciation and isotope evidence to identify plausible exposure media and pathway priorities in a potentially vulnerable worker population. The contribution of this study is therefore to integrate paired biomarkers, local foods, work and indoor dust, Hg speciation and hair Hg isotopes within the same dismantling-worker cohort to support pathway-priority screening. Specifically, we measured THg, MeHg and IHg in human biomarkers and dietary samples, THg in urine and work and indoor dust, and Hg isotopes in hair. The study was designed as an exploratory pathway-oriented biomonitoring assessment, not as a definitive source-apportionment or population-level exposure model. The conceptual design linking biomarkers, exposure matrices, Hg speciation and isotope evidence is summarized in Figure 1.

## 2. Materials and Methods

### 2.1. Study Design and Participants

The current dataset did not include a matched external control group or complete paired samples for every environmental matrix. Accordingly, the study was designed as an exploratory pilot assessment intended to identify exposure-pathway signals and inform future sampling priorities, rather than to estimate population-level effect sizes or make generalizable causal claims.

### 2.2. Sample Collection and Preparation

Hair samples were labeled H1–H18, blood samples B1–B18 and urine samples U1–U18 in the laboratory dataset. Food samples were labeled as meat (M), vegetables (V), rice (R) and fish (F). Fish and meat were purchased from markets near the workers’ residential area, whereas vegetables and rice were collected from workers’ vegetable gardens and family farmland. Dust A refers to work dust collected from e-waste dismantling-related work environments, whereas Dust B refers to indoor dust collected from living environments; both dust types were collected as settled dust by gently sweeping surfaces with a fine pig-bristle brush and sealing the material in polyethylene bags. For hair sampling and pretreatment, scalp hair was collected using stainless-steel scissors; dyed or bleached hair was excluded where identified. Hair samples were stored in clean bags, cut into small fragments, washed using an acetone–water–acetone sequence recommended for trace-element hair analysis, and dried in a dust-free environment before digestion or extraction. The individual-level biomarker, dust and hair isotope dataset is provided in Supplemental Table S1. This washing procedure reduces but does not fully prove removal of externally adsorbed particulate Hg; therefore, the hair IHg fraction is interpreted cautiously. Urine samples were collected in pre-cleaned polypropylene tubes and preserved by adding trace-metal-grade HNO_3_ to approximately 10% of the total volume, followed by sealed transport and storage at 4 °C until analysis. Concentrations are reported as μg/kg sample weight for solid matrices, μg/L for blood and μg/L for urine.

### 2.3. Mercury Concentration and Speciation Analysis

THg was measured in all matrices. MeHg was measured in hair, blood and food samples where sufficient material was available, and IHg was calculated as THg minus MeHg. The analytical workflow followed national food, environmental and biomonitoring methods where applicable, including GB 5009.17-2021, GB/T 17132-1997, HJ 1268-2022, HJ 1269-2022, HJ 977-2018 and HJ 910-2017 [23,24,25,26,27,28]; the full standard titles and method-performance details are provided in Supplemental Table S2. For urine THg, the analytical protocol followed previously established urine Hg biomonitoring methods, in which 0.2–3 mL of urine was digested with 5 mL of HNO_3_:H_2_SO_4_ mixture (4:1, *v*/*v*) at 95 °C for 3 h and determined by cold-vapor atomic fluorescence spectroscopy following USEPA Method 1631E [5]. THg in hair, blood, food and dust was determined by a DMA-80 direct mercury analyzer (Milestone Srl, Sorisole, Italy) based on thermal decomposition, gold amalgamation and atomic fluorescence detection. Urine THg was determined separately after acid digestion by cold-vapor atomic fluorescence spectroscopy. MeHg was determined by gas chromatography-cold vapor atomic fluorescence spectrometry (GC-CVAFS) using a Tekran 2700 mercury analyzer (Tekran Instruments Corporation, Toronto, ON, Canada) after species separation. IHg was calculated as THg minus MeHg. Matrix-specific LOD/LOQ and method-performance information is summarized in Supplemental Table S2.

### 2.4. Mercury Isotope Analysis

Hair Hg isotope compositions were measured using multicollector inductively coupled plasma mass spectrometry (MC-ICP-MS; Nu Plasma 1700, Nu Instruments, Wrexham, United Kingdom). The MC-ICP-MS was coupled with a CETAC Aridus II desolvating nebulizer (Teledyne CETAC Technologies, Omaha, NE, USA) for Tl introduction and a PerkinElmer FIAS-400 cold-vapor generation system (PerkinElmer, Waltham, MA, USA) for Hg^0^ generation. Hg isotope compositions were reported relative to NIST SRM 3133 (National Institute of Standards and Technology, Gaithersburg, MD, USA). Mass-dependent fractionation was expressed as δ^202^Hg, and odd-mass mass-independent fractionation was expressed as Δ^199^Hg and Δ^201^Hg. Instrumental mass bias was corrected using standard-sample bracketing and Tl internal normalization with NIST SRM 997 (National Institute of Standards and Technology, Gaithersburg, MD, USA). Sample and standard Hg concentrations were matched within approximately 10% where possible, and the introduction system was rinsed with 10% HNO_3_ between measurements to reduce memory effects. Mercury isotope ratios were calculated using the conventional delta notation relative to NIST SRM 3133. Odd-mass mass-independent fractionation was calculated as Δ^199^Hg = δ^199^Hg—0.252 × δ^202^Hg and Δ^201^Hg = δ^201^Hg—0.752 × δ^202^Hg. Analytical uncertainty was reported as two standard deviations (2SD) based on repeated measurements of in-house standards, certified reference materials and/or duplicate sample digestions within the analytical session. For samples without replicate isotope measurements, the external reproducibility of the nearest bracketing quality-control material was used as the minimum analytical uncertainty. Sample Hg concentrations and acid matrices were matched to NIST SRM 3133, and UM-Almaden secondary standard solutions diluted to 1 ng/mL Hg in 10% acid were analyzed using the same analytical treatment.

### 2.5. Quality Assurance and Quality Control

Quality assurance and quality control included procedural blanks, reagent blanks, blank spikes, matrix spikes, duplicate samples, certified reference materials and sample-standard bracketing. Procedural blanks were processed together with each batch of biological, food and dust samples to monitor potential contamination during digestion, extraction and instrumental analysis. Blank signals were below the method detection limit or negligible relative to the corresponding sample signals, and blank correction was applied where appropriate. Method-performance parameters, urine CRM recoveries, duplicate precision and isotope analytical uncertainty are summarized in Supplemental Table S2 and were interpreted with reference to established Hg speciation, urine biomonitoring and isotope protocols [5,16,17]. For Hg isotope QA/QC, UM-Almaden yielded δ^202^Hg = 0.56 ± 0.10‰, Δ^199^Hg = 0.04 ± 0.04‰, Δ^200^Hg = 0.02 ± 0.06‰ and Δ^201^Hg = 0.01 ± 0.06‰ (mean ± 2SD, *n* = 12), while BCR482 yielded δ^202^Hg = 1.53 ± 0.18‰, Δ^19^9Hg = 0.61 ± 0.14‰, Δ^200^Hg = 0.04 ± 0.04‰ and Δ^201^Hg = 0.56 ± 0.12‰ (mean ± 2SD, *n* = 8), consistent with published values.

### 2.6. Statistical Analysis

Data were summarized using medians and ranges because of the small sample size and non-normal distributions. Consistent with previous pathway-oriented e-waste exposure assessments, the analysis emphasized within-study matrix contrasts, pathway plausibility and comparison with published biomonitoring or environmental data rather than formal control-area inference. Spearman rank correlations were calculated only as exploratory analyses for THg concentrations among matrices when at least four paired observations were available, and each reported correlation is accompanied by the actual paired sample size. Correlations were not interpreted as evidence of causality and were not emphasized when paired n was very small. For hair isotope interpretation, the coupling between Δ^199^Hg and Δ^201^Hg was assessed using Spearman correlation and ordinary least-squares regression, and isotope patterns were compared qualitatively with published human biomonitoring studies. No imputation was performed for missing or QC-excluded speciation percentages. Statistical analyses were performed using Python 3.14 software (Python Software Foundation, Wilmington, DE, USA). The exploratory correlation matrix is provided in Supplemental Table S3.

## 3. Results

### 3.1. Sample Availability and Hg Concentrations

The analytical dataset included 18 participants. Hair THg and hair speciation were available for all 18 participants. Blood THg was available for 15 participants, and urine THg for 13 participants, reflecting incomplete biological sample coverage. Dietary sample coverage was highest for rice (*n* = 17) and lowest for fish (*n* = 12). Work dust (Dust A) was available for five participant-associated observations, and indoor dust (Dust B) for nine observations. These sample numbers are small, but the paired design allows a descriptive comparison of internal biomarkers with plausible dietary, work-dust and indoor-dust exposure matrices. The individual-level biomarker, dust and hair isotope dataset is provided in Supplemental Table S1.

**Figure 2:**
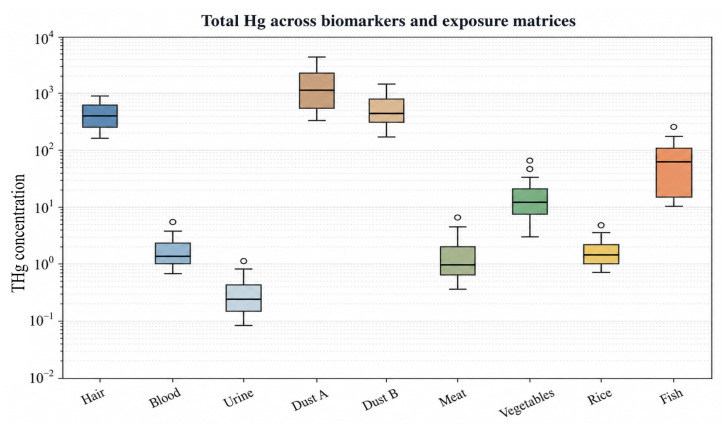
Distributions of THg concentrations in human biomarkers, work and indoor dust and dietary matrices. The y-axis is log-scaled to show low-concentration biological samples and high-concentration dust samples in the same panel. Dust A denotes work dust, and Dust B denotes indoor dust. Values are plotted in matrix-specific units (μg/kg for solid matrices, μg/L for blood and urine).

Median THg concentrations in human biomarkers were 403 μg/kg in hair, 1.60 μg/L in blood and 0.438 μg/L in urine (Table 1; Figure 2). Hair THg ranged from 229 to 778 μg/kg, indicating moderate inter-individual variation but no extremely high hair Hg burden in this small cohort. Among dietary matrices, fish had the highest median THg concentration (81.5 μg/kg), followed by vegetables (15.0 μg/kg), rice (2.97 μg/kg) and meat (2.11 μg/kg). Work and indoor dust showed the greatest THg enrichment, with median concentrations of 1605 and 755 μg/kg in the two dust groups, and the maximum value of 7222 μg/kg occurred in work dust (Dust A). Because the upper hair THg range overlapped with the lower part of the work- and indoor-dust distributions (Figure 2), these matrices should not be treated as completely separated statistical distributions. At the median/matrix level, the pattern nevertheless indicates lower biological-marker concentrations, intermediate dietary concentrations dominated by fish and vegetables, and higher THg reservoirs in work and indoor dust.

### 3.2. Mercury Speciation

**Figure 3:**
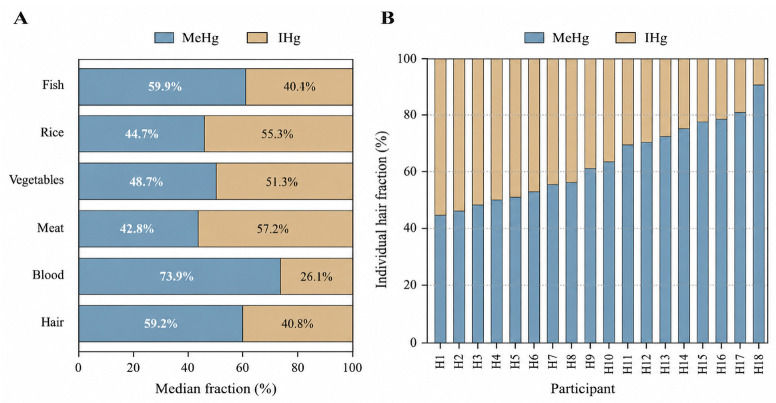
Hg speciation patterns. (**A**) Median MeHg and IHg fractions by matrix after quality-control flagging. (**B**) Participant-level hair MeHg and IHg fractions, sorted by MeHg proportion.

Hg speciation provided additional information beyond THg. Hair Hg was dominated by MeHg, with a median MeHg proportion of 59.2% (Table 2; Figure 3). This pattern is important because hair in general populations with mainly fish-derived MeHg exposure often contains a much larger MeHg fraction; a substantial IHg component can indicate either external contamination, environmental IHg exposure, or in vivo demethylation and incorporation of inorganic Hg. Blood showed a higher median MeHg proportion (73.9%), consistent with the role of recent dietary MeHg exposure in blood Hg. In food matrices, median MeHg proportions were 59.9% in fish, 48.7% in vegetables, 44.7% in rice and 42.8% in meat after excluding quality-control flagged values with MeHg greater than THg. Three low-concentration food samples were assigned QC flags because MeHg slightly exceeded THg or because calculated IHg was negative. These inconsistencies occurred in samples with low absolute Hg concentrations close to the method quantification range, where small differences between THg and MeHg measurements can produce unstable percentage estimates. Therefore, these observations were interpreted as analytical uncertainty near the detection range rather than evidence for MeHg fractions greater than 100%. Excluding these flagged samples did not change the overall matrix-level pattern: fish remained the food category with the highest THg and MeHg concentrations, whereas hair and blood retained mixed but MeHg-dominant speciation profiles.

The individual hair speciation profile showed that most participants had mixed MeHg-IHg composition rather than a uniformly MeHg-dominated pattern (Figure 3). This result supports a conservative interpretation: fish and other foods likely contributed MeHg, while dust or other e-waste-related environmental contact may have contributed inorganic Hg or external hair contamination. Because hair washing and external contamination controls were not fully documented in the available dataset, the IHg signal should be interpreted cautiously.

### 3.3. Hair Hg Isotope Signatures

**Figure 4:**
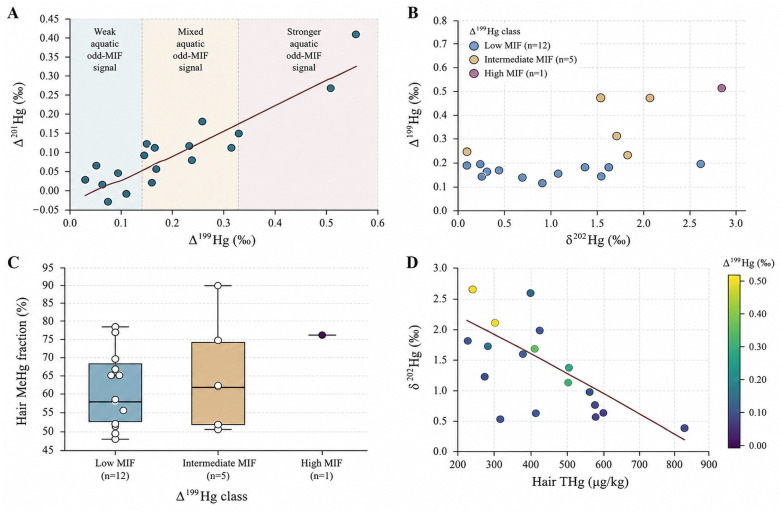
Literature-informed interpretation of hair Hg isotope signatures. (**A**) Δ^199^Hg-Δ^201^Hg coupling, with shaded domains representing weak, mixed and stronger aquatic odd-MIF signals. (**B**) δ^202^Hg versus Δ^199^Hg classified by Δ^199^Hg domain. (**C**) Hair MeHg fraction by Δ^199^Hg class. (**D**) Inverse relationship between hair THg and δ^202^Hg, with point color showing Δ199Hg. Solid lines in panels (**A**,**D**) indicate fitted trend lines; colors distinguish isotope-domain or Δ199Hg classes.

Hair δ^202^Hg ranged from 0.402 to 2.580‰, while Δ^199^Hg ranged from 0.084 to 0.532‰, and Δ^201^Hg from −0.012 to 0.461‰ (Supplemental Table S4; Figure 4). Δ^199^Hg and Δ^201^Hg were positively coupled (Spearman ρ = 0.78, *p* < 0.001; ordinary least-squares slope = 0.78, R^2^ = 0.85), supporting a coherent odd-mass MIF signal rather than random analytical scatter. However, most samples had modest Δ^199^Hg values: 12 of 18 samples were below 0.20‰, five were between 0.20 and 0.50‰, and only one exceeded 0.50‰. Hair THg was inversely associated with δ^202^Hg (Spearman ρ = −0.59, *p* = 0.010), whereas MeHg percentage was not associated with Δ^199^Hg (ρ = 0.00, *p* = 0.990). These patterns suggest that increasing hair THg did not simply reflect greater incorporation of a single fish-derived MeHg source. The H8 isotope values (Δ^199^Hg = 0.137‰ and Δ201Hg = −0.012‰) were retained after rechecking the original dataset; the negative Δ^201^Hg value is close to the isotope analytical uncertainty and was therefore not treated as an exclusion criterion.

The participant-level isotope-speciation map is provided in Supplemental Figure S1. The literature comparison places the present isotope results between two end-member patterns (Supplemental Figure S2). Fish- or seafood-dominated MeHg exposure commonly produces clearly positive odd-MIF in hair, and diet-to-hair δ^202^Hg offsets of approximately 1.7–2.2‰ have been reported in fish-consuming populations [7,19,20]. In contrast, rice and terrestrial dietary sources generally show smaller or near-zero MIF signatures compared with aquatic MeHg sources [18,21,22]. The Qingyuan samples had lower median Δ^199^Hg than typical marine-fish consumer groups but higher and more variable values than a purely near-zero-MIF inorganic source would predict. This supports a mixed-exposure interpretation rather than a single-source interpretation.

### 3.4. Exploratory Relationships Among Matrices

Exploratory Spearman correlations were used only to screen for patterns that could guide future studies, and the full correlation matrix is provided in Supplemental Table S3. Hair THg showed a positive exploratory correlation with indoor dust THg in the limited paired dataset (rho = 0.65, *p* = 0.058, *n* = 9), whereas hair THg was negatively associated with fish THg (rho = −0.45, *p* = 0.142, *n* = 12). Meat THg was moderately correlated with indoor dust (Dust B) THg (rho = 0.62, *p* = 0.101, *n* = 8) and vegetables THg (rho = 0.49, *p* = 0.064, *n* = 15). Because many pairwise comparisons involved very small paired sample sizes and were not adjusted for multiple testing, these correlations should be interpreted as hypothesis-generating rather than confirmatory evidence.

### 3.5. Simplified Exposure Pathway Ranking

To make the pathway interpretation explicit without over-fitting this pilot dataset, exposure pathways were ranked qualitatively using matrix THg/MeHg levels, Hg speciation, hair isotope consistency and biomarker support. This ranking is intended to identify priority pathways for future controlled exposure and risk-assessment studies rather than to estimate absorbed dose (Table 3).

## 4. Discussion

This study is best interpreted as a pilot pathway-oriented human exposure assessment rather than a controlled comparison between exposed and unexposed populations. The participant-level isotope-speciation map and literature-range comparison are provided in Supplemental Figures S1 and S2, respectively. This framing follows previous e-waste exposure assessments from South China, which used multi-matrix data from food, dust and water to identify important exposure media and pathway priorities even when a formal control group was not available for every matrix [8,11]. Under this framework, the central finding is not that the workers had unusually high Hg exposure, but that the available matrices point to a mixed exposure pattern that is relevant for future quantitative human exposure and risk assessment. Hair THg was below the commonly used 1 μg/g reference level for MeHg exposure [29], and blood and urine THg concentrations were also low relative to levels typically associated with clinically significant Hg poisoning or high occupational exposure [1]. Therefore, the present data should not be interpreted as evidence of excessive Hg exposure. Instead, the main implication is that even at low-to-moderate biomarker concentrations, paired speciation and isotope information can reveal pathway patterns that are not apparent from THg alone.

### 4.1. Concentration Evidence

The concentration data provide the first line of evidence. Fish contained the highest THg among the food categories and also had the highest median MeHg proportion, consistent with the established role of fish as a MeHg source and with previous e-waste-region exposure assessments emphasizing rice and fish consumption as relevant dietary pathways [10]. Work and indoor dust contained much higher THg than foods and biological matrices, indicating that a local environmental Hg reservoir was present in at least some sampled environments. These dust data do not prove uptake or distinguish residential from occupational exposure, but they justify considering non-dietary Hg contact as a plausible contributor in this e-waste setting, consistent with previous South China studies identifying house or indoor dust as an important exposure medium [8,11].

### 4.2. Speciation Evidence

Hair speciation provides the second line of evidence. The median MeHg fraction in hair was approximately 59%, lower than would be expected for a clean dietary MeHg-dominated exposure scenario. The remaining IHg fraction may reflect direct incorporation of inorganic Hg, external adsorption of particulate Hg onto hair, demethylation of MeHg, or a mixture of these mechanisms. Because the dataset lacks detailed hair-washing validation, segmental hair analysis and paired dust isotope data, the IHg signal should not be interpreted as internal dose alone. Nevertheless, the combination of moderate hair THg, substantial hair IHg and elevated work and indoor dust THg is more consistent with mixed exposure than with a purely fish-derived MeHg pattern. This interpretation is supported by previous hair speciation and isotope studies showing that hair THg alone can obscure differences in Hg chemical form and exposure source, particularly when occupational, inorganic or externally adsorbed Hg contributions are present [7,30,31].

### 4.3. Isotope Evidence

The isotope data provide the third line of evidence. Stable Hg isotopes have been used to trace Hg pathways across environmental matrices and human biomarkers in contaminated settings [20,32]. In the present study, positive Δ^199^Hg and Δ^201^Hg values in hair are consistent with an aquatic dietary MeHg contribution, and the coupled Δ^199^Hg–Δ^201^Hg relationship suggests that the MIF signal is meaningful. However, the magnitude of the signal is important. The median Δ^199^Hg was only 0.162‰, lower than values reported for several marine-fish or seafood-consuming populations [19,20], and 12 of 18 hair samples were below 0.20‰. This pattern is compatible with an aquatic-food contribution diluted by weak-MIF or near-zero-MIF Hg sources, including rice, terrestrial dietary inputs, inorganic Hg or externally adsorbed particulate Hg [18,21,22], but it does not allow quantitative source apportionment. A participant-level isotope-speciation map further illustrates this qualitative separation between stronger fish-type MeHg signals and weaker mixed or inorganic/particulate Hg signatures (Supplemental Figure S1).

The comparison with occupational and compound-specific isotope studies strengthens this cautious interpretation. Occupational inorganic-Hg settings can show low MeHg percentages and low-to-moderate hair Δ^199^Hg, indicating that hair THg may include substantial inorganic or externally adsorbed Hg [31]. Conversely, compound-specific studies show that bulk-hair THg isotope values can obscure differences between MMHg and IHg pools; therefore, quantitative source apportionment would require compound-specific Hg isotope analysis, direct isotope measurements of local fish, rice, vegetables and dust, or both [16,17].

**Figure 5:**
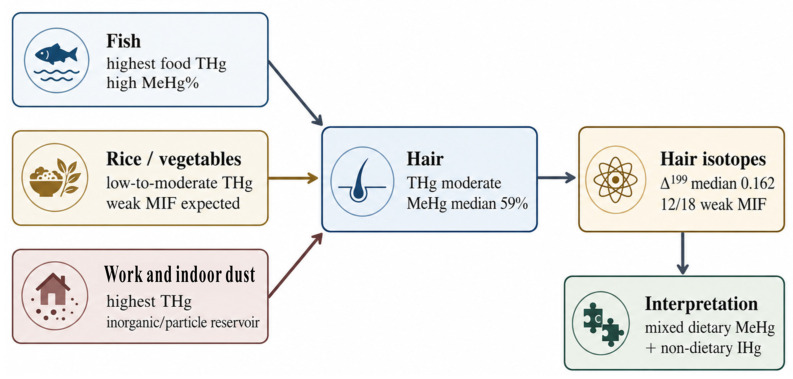
Synthesis of the mixed-exposure hypothesis for pathway-oriented human exposure assessment. The diagram summarizes how dietary matrices, work dust, indoor dust, hair speciation and hair isotope evidence converge on a qualitative interpretation that is consistent with mixed exposure, not quantitative apportionment.

Compared with previous e-waste studies, this work should be positioned as complementary rather than first-of-its-kind. Earlier studies from Qingyuan and related South China e-waste areas used multi-matrix sampling of food, house dust, water and indoor dust to evaluate exposure media and pathway contributions, showing that such pathway-oriented designs can produce publishable evidence even without a complete biomonitoring control structure for every endpoint [8,11]. The present study extends that local multi-matrix logic to Hg by linking paired biomarkers, local foods, work and indoor dust, Hg speciation and hair isotopes in a dismantling-worker cohort. Its contribution is therefore pathway screening and method demonstration, not definitive attribution of e-waste-derived Hg uptake. Figure 5 summarizes how the dietary, dust, biomarker-speciation and isotope evidence converge on the mixed-exposure interpretation.

The limitations should be stated explicitly but interpreted in relation to the pathway-characterization aim. The study lacks an external control group, has only 18 participants, and has incomplete blood, urine, fish and dust coverage. These limitations prevent between-area causal inference, quantitative source apportionment and formal health-risk estimation, but they do not preclude descriptive identification of plausible exposure media within the study area. Questionnaire variables such as fish consumption frequency, occupational duration, use of personal protective equipment, smoking, dental amalgam, hair treatment and exact residential proximity were not available in the current dataset. Urinary THg was not adjusted for creatinine or specific gravity, and therefore urine dilution may have contributed to inter-individual variability. Work and indoor dust were analyzed only for THg; without dust Hg speciation or isotope measurements, the contribution of dust-derived IHg to internal exposure cannot be quantified. Several low-concentration food samples showed MeHg slightly greater than THg or negative calculated IHg. These observations were explicitly flagged and excluded from percentage-based speciation summaries. Because they occurred near the method quantification range, they most likely reflect analytical uncertainty amplified by ratio calculation rather than true MeHg fractions exceeding 100%. The appropriate conclusion is therefore preliminary and pathway-focused: the dataset supports mixed exposure hypotheses that justify larger targeted exposure and risk-assessment studies, but it does not establish the magnitude of e-waste-attributable Hg uptake. In addition, the dust samples represented swept settled dust rather than a defined respirable or ingestible particle-size fraction, so the dust results should be interpreted as environmental reservoir indicators rather than direct personal exposure measurements.

## 5. Conclusions

This pilot pathway-oriented biomonitoring assessment indicates that the observed biomarker, work-dust, indoor-dust, dietary and hair-isotope patterns are consistent with mixed dietary MeHg and non-dietary IHg contributions among e-waste dismantling workers in Qingyuan. Fish-MeHg ranked as the most plausible dietary MeHg pathway, while work and indoor dust represented the strongest non-dietary Hg reservoirs. Vegetable and rice pathways appeared secondary in this dataset. Hair Hg isotope signatures were compatible with an aquatic dietary contribution but did not permit quantitative source apportionment. The findings should be interpreted as preliminary because of the small sample size, incomplete sample coverage and limited source-isotope information. Nevertheless, the study demonstrates the feasibility and value of integrating multi-matrix biomonitoring, Hg speciation and Hg isotopes to identify exposure-pathway priorities in e-waste-impacted settings.

## Figures and Tables

**Table 1:** Sample coverage and THg concentrations by matrix.

Matrix	Sample IDs (n)	THg (n)	THg Median (μg/kg Solid; μg/L Blood; μg/L Urine)	THg Range (μg/kg Solid; μg/L Blood; μg/L Urine)
Hair	18	18	403	229–778
Blood	16	15	1.60	1.02–3.08
Urine	14	13	0.44	0.15–0.91
Work dust (Dust A)	5	5	1605	566–7222
Indoor dust (Dust B)	9	9	755	335–2275
Meat	15	15	2.11	0.35–6.78
Vegetables	16	16	15.0	3.13–67.1
Rice	17	17	2.97	0.76–5.11
Fish	12	12	81.5	10.3–255

**Table 2:** Hg speciation summary for matrices with MeHg data.

Matrix	MeHg (n)	MeHg Median (μg/kg Solid; μg/L Blood)	MeHg Median (%)
Hair	18	248	59.2
Blood	15	1.24	73.9
Meat	14	0.70	42.8
Vegetables	13	6.68	48.7
Rice	17	1.22	44.7
Fish	12	49.8	59.9

Note: MeHg% was calculated as MeHg/THg × 100. Samples with MeHg exceeding THg or negative calculated IHg were assigned QC flags and excluded from MeHg% summaries. These flagged observations occurred in low-concentration food samples near the method quantification range and were not interpreted as biological evidence of MeHg fractions greater than 100%.

**Table 3:** Simplified qualitative ranking of plausible Hg exposure pathways.

Rank	Pathway	Key Evidence and Interpretation
1	Fish-MeHg	Highest food THg median (81.5 μg/kg) and MeHg median (49.8 μg/kg); positive but modest hair odd-MIF. Strongest dietary MeHg signal; contribution plausible, but intake not quantified.
2	Dust-IHg/Particulate Hg	Work dust THg median 1605 μg/kg; indoor dust THg median 755 μg/kg; hair IHg median 145 μg/kg. Strong environmental reservoir; uptake cannot be quantified without dust speciation/isotopes.
3	Vegetable-MeHg/mixed Hg	Vegetable THg median 15.0 μg/kg and MeHg median 6.7 μg/kg, with several low-Hg level QC flags. Secondary dietary pathway; ratio-based speciation should be interpreted cautiously.
4	Rice-MeHg/mixed Hg	Rice THg median 2.97 μg/kg and MeHg median 1.22 μg/kg. Low-concentration dietary pathway in this cohort; still useful for local diet context.
5	Meat-MeHg/mixed Hg	Meat THg median 2.11 μg/kg and MeHg median 0.70 μg/kg. Lower-priority pathway based on concentration and matrix ranking.

Note: Ranking reflects relative pathway plausibility in this pilot dataset. It does not represent quantitative daily intake or source apportionment because matrix-specific intake rates, absorption efficiencies and source-isotope endmembers were unavailable.

## Data Availability

The original contributions presented in this study are included in the Supplementary Material. Further inquiries can be directed to the author.

## Supplementary Materials

The following supporting information can be downloaded at: https://www.mdpi.com/article/10.3390/toxics14070584/s1, Figure S1. Participant-level isotope-speciation map used for discussion. Shaded fields show interpretive domains derived from the literature: high MeHg fraction with stronger odd-MIF is more consistent with fish-type MeHg exposure, whereas lower MeHg fractions and weak-to-moderate odd-MIF indicate possible inorganic or external particulate Hg contributions. The classification is qualitative; Figure S2. Literature ranges used to contextualize the present hair isotope and speciation results. The Qingyuan workers overlap partly with mixed occupational or inland exposure settings but have lower Δ^199^Hg than strongly seafood-dominated populations; Table S1. Individual-level biomarker, dust and hair isotope dataset; Table S2. Analytical standards, method-performance parameters and quality-control information for mercury analyses; Table S3. Exploratory Spearman correlation matrix among THg concentrations in paired matrices; Table S4. Summary of hair Hg isotope and speciation data for primary hair samples (*n* = 18).

## References

[1] ATSDR (2024). Toxicological Profile for Mercury.

[2] O’Connor D., Hou D., Ok Y.S., Mulder J., Duan L., Wu Q., Wang S., Tack F.M., Rinklebe J. (2019). Mercury speciation, transformation, and transportation in soils, atmospheric flux, and implications for risk management: A critical review. Environ. Int..

[3] WHO Regional Office for Europe (2020). Human Biomonitoring: Facts and Figures on Mercury.

[4] Wang Y., Li L., Yao C., Tian X., Wu Y., Xie Q., Wang D. (2021). Mercury in human hair and its implications for health investigation. Curr. Opin. Environ. Sci. Health.

[5] Du B., Yin R., Fu X., Li P., Feng X., Maurice L. (2021). Use of mercury isotopes to quantify sources of human inorganic mercury exposure and metabolic processes in the human body. Environ. Int..

[6] Sherman L.S., Blum J.D., Franzblau A., Basu N. (2013). New insight into biomarkers of human mercury exposure using naturally occurring mercury stable isotopes. Environ. Sci. Technol..

[7] Laffont L., Sonke J.E., Maurice L., Monrroy S.L., Chincheros J., Amouroux D., Behra P. (2011). Hg speciation and stable isotope signatures in human hair as a tracer for dietary and occupational exposure to mercury. Environ. Sci. Technol..

[8] He C.-T., Zheng X.-B., Yan X., Zheng J., Wang M.-H., Tan X., Qiao L., Chen S.-J., Yang Z.-Y., Mai B.-X. (2017). Organic contaminants and heavy metals in indoor dust from e-waste recycling, rural, and urban areas in South China: Spatial characteristics and implications for human exposure. Ecotoxicol. Environ. Saf..

[9] Ni W., Chen Y., Huang Y., Wang X., Zhang G., Luo J., Wu K. (2014). Hair mercury concentrations and associated factors in an electronic waste recycling area, Guiyu, China. Environ. Res..

[10] Tang W., Cheng J., Zhao W., Wang W. (2015). Mercury levels and estimated total daily intakes for children and adults from an electronic waste recycling area in Taizhou, China: Key role of rice and fish consumption. J. Environ. Sci..

[11] Zheng J., Chen K.-H., Yan X., Chen S.-J., Hu G.-C., Peng X.-W., Yuan J.-G., Mai B.-X., Yang Z.-Y. (2013). Heavy metals in food, house dust, and water from an e-waste recycling area in South China and the potential risk to human health. Ecotoxicol. Environ. Saf..

[12] Zheng J., Luo X.-J., Yuan J.-G., He L.-Y., Zhou Y.H., Luo Y., Chen S.-J., Mai B.-X., Yang Z.-Y. (2011). Heavy metals in hair of residents in an e-waste recycling area, South China: Contents and assessment of bodily state. Arch. Environ. Contam. Toxicol..

[13] Gomes L.d.R., Martins C., Fernandes M., Viegas S. (2024). Unveiling mercury exposure sources in e-waste recycling with biomonitoring: A Portuguese case study. Eur. J. Public Health.

[14] Leese E., Verdonck J., Porras S.P., Airaksinen J., Duca R.C., Galea K.S., Godderis L., Janasik B., Mahiout S., Martins C. (2025). HBM4EU e-waste study: Occupational exposure assessment to chromium, cadmium, mercury and lead during e-waste recycling. Environ. Res..

[15] Scheepers P.T.J., Duca R.C., Galea K.S., Godderis L., Hardy E., Knudsen L.E., Leese E., Louro H., Mahiout S., Ndaw S. (2021). HBM4EU occupational biomonitoring study on e-waste: Study protocol. Int. J. Environ. Res. Public Health.

[16] Wang B., Yang S., Li P., Qin C., Wang C., Ali M.U., Yin R., Maurice L., Point D., Sonke J.E. (2023). Trace mercury migration and human exposure in typical mercury-emission areas by compound-specific stable isotope analysis. Environ. Int..

[17] Yang S., Wang B., Qin C., Yin R., Li P., Liu J., Point D., Maurice L., Sonke J.E., Zhang L. (2021). Compound-specific stable isotope analysis provides new insights for tracking human monomethylmercury exposure sources. Environ. Sci. Technol..

[18] Du B., Feng X., Li P., Yin R., Yu B., Sonke J.E., Guinot B., Anderson C.W.N., Maurice L. (2018). Use of mercury isotopes to quantify mercury exposure sources in inland populations, China. Environ. Sci. Technol..

[19] Laffont L., Sonke J.E., Maurice L., Hintelmann H., Pouilly M., Bacarreza Y.S., Perez T., Behra P. (2009). Anomalous mercury isotopic compositions of fish and human hair in the Bolivian Amazon. Environ. Sci. Technol..

[20] Li M., Sherman L.S., Blum J.D., Grandjean P., Mikkelsen B., Weihe P., Sunderland E.M., Shine J.P. (2014). Assessing sources of human methylmercury exposure using stable mercury isotopes. Environ. Sci. Technol..

[21] Li P., Du B., Maurice L., Laffont L., Lagane C., Point D., Sonke J.E., Yin R., Lin C.-J., Feng X. (2017). Mercury isotope signatures of methylmercury in rice samples from the Wanshan mercury mining area, China: Environmental implications. Environ. Sci. Technol..

[22] Rothenberg S.E., Yin R., Hurley J.P., Krabbenhoft D.P., Ismawati Y., Hong C., Donohue A. (2017). Stable mercury isotopes in polished rice (*Oryza sativa* L.) and hair from rice consumers. Environ. Sci. Technol..

[23] (2021). National Food Safety Standard—Determination of Total Mercury and Organic Mercury in Foods.

[24] (1997). Soil Quality—Determination of Total Mercury—Cold Atomic Absorption Spectrophotometry.

[25] (2022). Water Quality—Determination of Methylmercury and Ethylmercury—Liquid Chromatography/Atomic Fluorescence Spectrometry.

[26] (2022). Soil and Sediment—Determination of Methylmercury and Ethylmercury—Purge and Trap/Gas Chromatography-Cold Vapor Atomic Fluorescence Spectrometry.

[27] (2018). Water Quality—Determination of Alkyl Mercury—Purge and Trap/Gas Chromatography-Cold Vapor Atomic Fluorescence Spectrometry.

[28] (2017). Ambient Air—Determination of Gaseous Mercury—Gold Amalgamation Collection and Analysis by Cold Vapor Atomic Absorption Spectrophotometry.

[29] US EPA (2001). Water Quality Criterion for the Protection of Human Health: Methylmercury.

[30] Kanwal S., Yamakawa A., Narukawa T., Yoshinaga J. (2019). Speciation and isotopic characterization of mercury detected at high concentration in Pakistani hair samples. Chemosphere.

[31] Sherman L.S., Blum J.D., Basu N., Rajaee M., Evers D.C., Buck D.G., Petrlik J., DiGangi J. (2015). Assessment of mercury exposure among small-scale gold miners using mercury stable isotopes. Environ. Res..

[32] Bonsignore M., Tamburrino S., Oliveri E., Marchetti A., Durante C., Berni A., Quinci E., Sprovieri M. (2015). Tracing mercury pathways in Augusta Bay (southern Italy) by total concentration and isotope determination. Environ. Pollut..

