# Supplementary material for: Pilot Multi-Matrix Biomonitoring of Mixed Mercury Exposure Pathways Among E-Waste Dismantling Workers in South China
Source: Toxics. 2026 Jul 2;14(7):584. doi: 10.3390/toxics14070584 (PMC13417384; doi:10.3390/toxics14070584)
Supplement: Supplementary file 1 [file toxics-14-00584-s001.zip › toxics-4354558-supplementary.pdf]

Supplemental Material

Pilot multi-matrix biomonitoring of mixed mercury exposure pathways among e-waste dismantling workers in South China

Qiyuan Lu

This supplemental material contains:

2 Supplemental Figures

4 Supplemental Tables

Supplemental Figure S1. Participant-level isotope-speciation map.

Supplemental Figure S2. Literature range comparison for hair $\Delta^{199}Hg$ and MeHg fraction. Literature ranges were compiled from studies cited in the main manuscript.

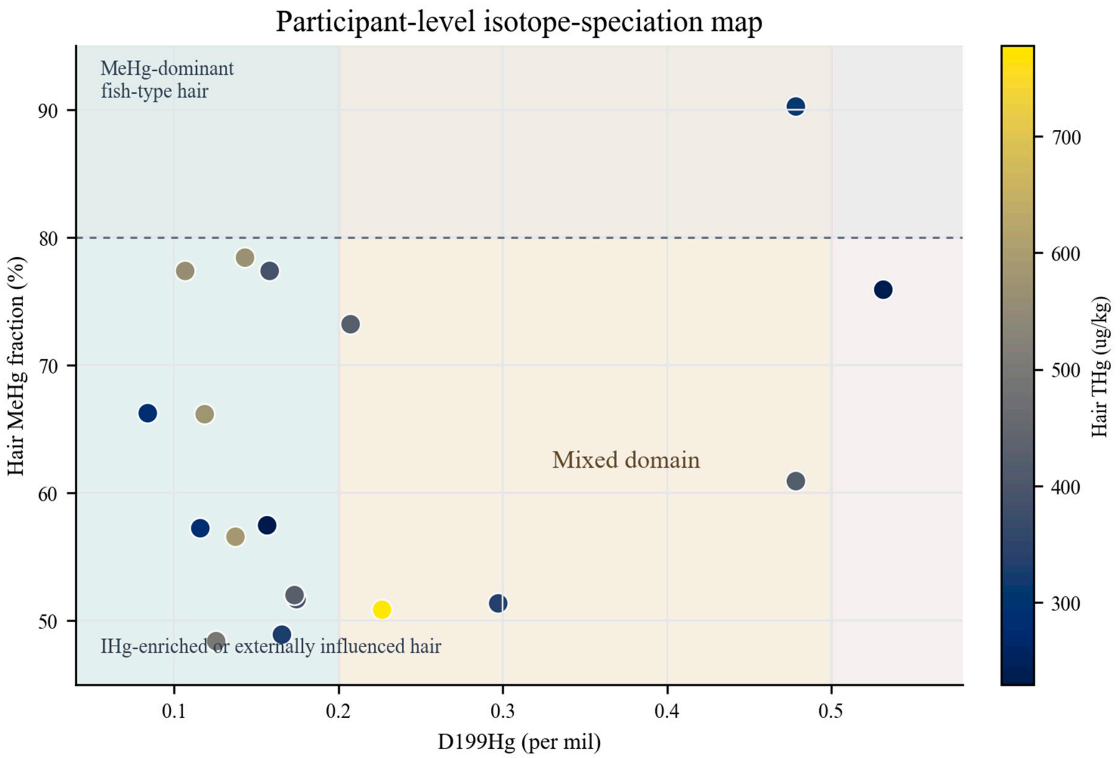

Figure S1. Participant-level isotope-speciation map used for discussion. Shaded fields show interpretive domains derived from the literature: high MeHg fraction with stronger odd-MIF is more consistent with fish-type MeHg exposure, whereas lower MeHg fractions and weak-to-moderate odd-MIF indicate possible inorganic or external particulate Hg contributions. The classification is qualitative.

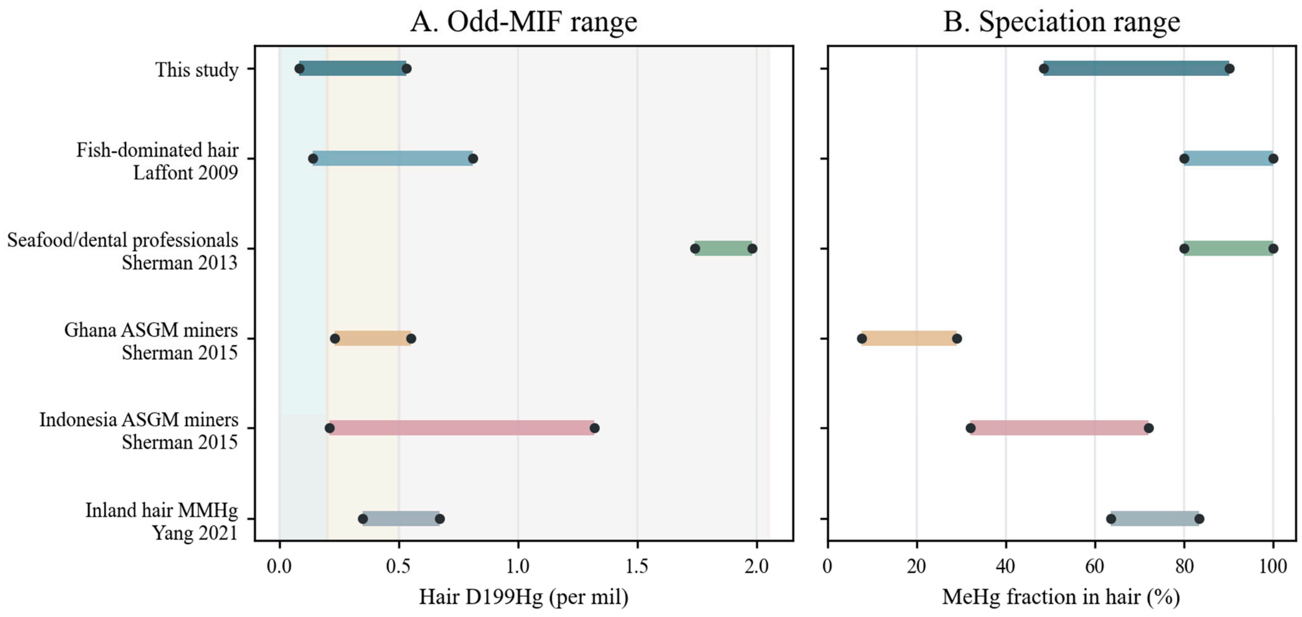

Figure S2. Literature ranges used to contextualize the present hair isotope and speciation results. The Qingyuan workers overlap partly with mixed occupational or inland exposure settings but have lower $\Delta^{199}Hg$ than strongly seafood-dominated populations.

Table S1. Individual-level Hg concentration, speciation, isotope and paired matrix dataset.

ID	Hair THg (µg/kg)	MeHg (µg/kg)	IHg (µg/kg)	MeHg (%)	Blood THg (µg/L)	Urine THg (µg/L)	Work dust THg (µg/kg)	Indoor dust THg (µg/kg)	δ^{202}Hg (‰)	Δ^{199}Hg (‰)	Δ^{201}Hg (‰)
H4	229.272	131.734	97.538	57.457	1.526	0.543	-	-	1.607	0.157	0.118
H15	232.847	176.724	56.123	75.897	1.377	0.438	-	-	2.580	0.532	0.461
H10	286.122	189.505	96.617	66.232	2.110	0.455	-	600.557	1.082	0.084	0.060
H13	290.678	166.347	124.331	57.227	1.419	0.169	-	538.212	1.535	0.116	0.056
H2	314.165	283.481	30.685	90.233	1.313	0.479	-	334.615	1.977	0.478	0.278
H14	329.156	160.953	168.203	48.899	1.261	0.377	-	754.523	0.402	0.166	0.123
H1	336.181	172.619	163.562	51.347	2.489	0.244	7221.515	2118.549	1.652	0.297	0.149
H11	386.577	299.084	87.492	77.367	1.601	0.176	-	400.264	1.450	0.158	0.100
H5	387.687	200.352	187.335	51.679	-	-	-	-	2.393	0.174	0.066
H3	418.888	255.168	163.721	60.915	2.383	-	-	-	1.511	0.478	0.278
H12	422.780	309.447	113.333	73.193	1.806	0.912	566.069	-	1.747	0.207	0.115
H6	426.353	221.681	204.673	51.995	-	0.146	4440.139	-	0.515	0.173	0.021
H7	496.360	240.188	256.173	48.390	3.082	0.639	-	828.591	1.216	0.125	0.023
H18	559.371	432.747	126.623	77.363	-	-	-	-	0.874	0.107	0.035
H17	571.598	448.157	123.441	78.404	2.466	-	-	-	0.682	0.143	0.097
H9	575.156	380.480	194.676	66.153	2.193	0.161	776.426	1436.197	0.522	0.119	0.025
H8	589.975	333.709	256.265	56.563	1.017	0.526	1604.681	2274.719	0.576	0.137	−0.012
H16	777.994	395.618	382.376	50.851	1.503	-	-	-	0.406	0.227	0.181

Note: - indicates unavailable measurements.

Food provenance: fish and meat were purchased from markets near the workers' residential area; vegetables and rice were collected from workers' vegetable gardens and family farmland. Dust A and Dust B were swept settled dust collected with a fine pig-bristle brush and stored in polyethylene bags. δ^{202}Hg denotes mass-dependent Hg isotope fractionation, and Δ^{199}Hg and Δ^{201}Hg denote odd-mass mass-independent Hg isotope fractionation.

Table S2. Analytical standards, method-performance parameters and quality-control information for mercury analyses.
Additional isotope QA/QC: UM-Almaden secondary standard solutions (1 ng/mL Hg in 10% acid) were analyzed with the same analytical treatment. Results were δ202Hg = 0.56 ± 0.10‰, Δ199Hg = 0.04 ± 0.04‰, Δ200Hg = 0.02 ± 0.06‰ and Δ201Hg = 0.01 ± 0.06‰ (mean ± 2SD, n = 12). BCR482 results were δ202Hg = 1.53 ± 0.18‰, Δ199Hg = 0.61 ± 0.14‰, Δ200Hg = 0.04 ± 0.04‰ and Δ201Hg = 0.56 ± 0.12‰ (mean ± 2SD, n = 8).

**Parameter**	**Matrix / material**	**Value**
THg LOD	Hair / fish	0.07 ng/g
THg LOD	Rice	0.03 ng/g
THg LOD	Comparable biological/food protocol	0.02 ng/g
MeHg LOD	Hair / fish / rice and related matrices	0.003–0.004 ng/g
Urine THg LOD	Human urine	0.1 μg/L
Estimated THg LOQ	Solid matrices, based on 10/3 × LOD	0.067–0.233 ng/g
Estimated MeHg LOQ	Based on 10/3 × LOD	0.010–0.013 ng/g
Estimated urine THg LOQ	Human urine, based on 10/3 × LOD	0.33 μg/L
MeHg recovery	CRMs, rice, fish, hair	93 ± 8%
MeHg extract purity	Digested solutions	94 ± 7%
THg / MeHg duplicate precision	Duplicate solid samples	RPD <10%
THg / MeHg CRM recovery	CRMs	>90%
Urine THg CRM recovery	ZK020-1, human urine, China CDC	94 ± 9%
Urine THg CRM recovery	ZK020-2, human urine, China CDC	95 ± 7%
Urine THg duplicate precision	Duplicate urine samples	RPD <10%
THg isotope digestion recovery	Digested samples	80–120%
δ^{202}Hg uncertainty	Reference standards	±0.08–0.10‰
Δ^{199}Hg uncertainty	Reference standards	±0.04–0.08‰
Δ^{201}Hg uncertainty	Reference standards	±0.06–0.12‰
Reference materials	CRMs / standards	TORT-2, ERM-CE464, BCR-482, NIES-13, ZK020-1, ZK020-2, UM-Almadén, NIST SRM 3133
Analytical standard	GB 5009.17-2021	National Food Safety Standard - Determination of Total Mercury and Organic Mercury in Foods; National Health Commission of the PRC and State Administration for Market Regulation: Beijing, China, 2021.

Analytical standard	GB/T 17132-1997	Soil Quality - Determination of Total Mercury - Cold Atomic Absorption Spectrophotometry; State Bureau of Technical Supervision: Beijing, China, 1997.
Analytical standard	HJ 1268-2022	Water Quality - Determination of Methylmercury and Ethylmercury - Liquid Chromatography / Atomic Fluorescence Spectrometry; Ministry of Ecology and Environment of the PRC: Beijing, China, 2022.
Analytical standard	HJ 1269-2022	Soil and Sediment - Determination of Methylmercury and Ethylmercury - Purge and Trap / Gas Chromatography-Cold Vapor Atomic Fluorescence Spectrometry; Ministry of Ecology and Environment of the PRC: Beijing, China, 2022.
Analytical standard	HJ 977-2018	Water Quality - Determination of Alkyl Mercury - Purge and Trap / Gas Chromatography-Cold Vapor Atomic Fluorescence Spectrometry; Ministry of Ecology and Environment of the PRC: Beijing, China, 2018.
Analytical standard	HJ 910-2017	Ambient Air - Determination of Gaseous Mercury - Gold Amalgamation Collection and Analysis by Cold Vapor Atomic Absorption Spectrophotometry; Ministry of Environmental Protection of the PRC: Beijing, China, 2017.

Table S3. Exploratory Spearman correlation matrix among THg concentrations in paired matrices.
Note: p values are exploratory two-sided p values based on Spearman rank correlations and are not adjusted for multiple comparisons. Approximate 95% CIs for Spearman rho were calculated using Fisher z transformation and are provided for descriptive uncertainty assessment only.

Matrix pair	Paired n	Spearman rho	p value	Approx. 95% CI for rho
Hair vs Blood	15	0.21	0.453	-0.34 to 0.65
Hair vs Urine	13	-0.08	0.795	-0.60 to 0.49
Hair vs Work dust	5	-0.30	0.624	-0.93 to 0.79
Hair vs Indoor dust	9	0.65	0.058	-0.02 to 0.92
Hair vs Meat	15	0.01	0.972	-0.50 to 0.52
Hair vs Vegetables	16	-0.24	0.371	-0.66 to 0.29
Hair vs Rice	17	-0.20	0.442	-0.62 to 0.31
Hair vs Fish	12	-0.45	0.142	-0.81 to 0.17
Blood vs Urine	12	-0.03	0.926	-0.59 to 0.55
Blood vs Work dust	4	0.40	0.600	-0.91 to 0.98
Blood vs Indoor dust	9	0.17	0.662	-0.56 to 0.75
Blood vs Meat	14	0.38	0.180	-0.19 to 0.76
Blood vs Vegetables	15	0.21	0.453	-0.34 to 0.65
Blood vs Rice	15	-0.50	0.058	-0.81 to 0.02
Blood vs Fish	10	0.15	0.679	-0.53 to 0.71
Urine vs Work dust	5	-0.50	0.391	-0.96 to 0.68
Urine vs Indoor dust	9	0.13	0.739	-0.58 to 0.73
Urine vs Meat	12	0.29	0.361	-0.34 to 0.74
Urine vs Vegetables	13	-0.19	0.534	-0.67 to 0.40
Urine vs Rice	13	0.25	0.410	-0.35 to 0.70
Urine vs Fish	10	0.16	0.659	-0.52 to 0.72
Work dust vs Meat	4	0.40	0.600	-0.91 to 0.98
Work dust vs Vegetables	5	0.60	0.285	-0.60 to 0.97
Work dust vs Rice	5	-0.30	0.624	-0.93 to 0.79
Work dust vs Fish	5	0.70	0.188	-0.48 to 0.98
Indoor dust vs Meat	8	0.62	0.101	-0.15 to 0.92
Indoor dust vs Vegetables	9	0.13	0.739	-0.58 to 0.73

Indoor dust vs Rice	9	0.22	0.570	-0.52 to 0.77
Indoor dust vs Fish	7	-0.14	0.765	-0.81 to 0.69
Meat vs Vegetables	15	0.49	0.064	-0.03 to 0.80
Meat vs Rice	15	0.14	0.619	-0.40 to 0.61
Meat vs Fish	10	0.45	0.192	-0.25 to 0.84
Vegetables vs Rice	16	0.36	0.171	-0.17 to 0.73
Vegetables vs Fish	11	0.18	0.596	-0.47 to 0.70
Rice vs Fish	11	-0.46	0.155	-0.83 to 0.19

Table S4. Summary of hair Hg isotope and speciation data for primary hair samples (n = 18).
Note for H8: Δ199Hg = 0.137‰ and Δ201Hg = -0.012‰ were retained after rechecking the original dataset; the Δ201Hg value is close to isotope analytical uncertainty.

**Variable**	**n**	**Mean**	**SD**	**Min**	**Median**	**Max**
THg (μg/kg)	18	423.953	146.292	229.272	403.288	777.994
MeHg (μg/kg)	18	266.555	99.218	131.734	247.678	448.157
IHg (μg/kg)	18	157.398	83.481	30.685	145.093	382.376
MeHg %	18	63.343	12.684	48.390	59.186	90.233
IHg %	18	36.657	12.684	9.767	40.814	51.610
δ^{202}Hg	18	1.263	0.676	0.402	1.333	2.580
Δ^{199}Hg	18	0.215	0.138	0.084	0.162	0.532
Δ^{201}Hg	18	0.121	0.118	−0.012	0.098	0.461
